# Diagnostic Utility of Pleural Fluid Lactate and Pleural Fluid-to-Serum Lactate Ratio in Differentiating Various Etiologies of Exudative Pleural Effusions: A Cross-Sectional Observational Study

**DOI:** 10.7759/cureus.107062

**Published:** 2026-04-14

**Authors:** Shweta Ambedare, Praveen Bharti, Sandeep Garg, Ekta Debnath, Amit Kumar, Ved Prakash Yadav, Tushar Ambedare

**Affiliations:** 1 General Medicine, Maulana Azad Medical College, Lok Nayak Hospital, New Delhi, IND; 2 Biochemistry, Maulana Azad Medical College, Lok Nayak Hospital, New Delhi, IND; 3 Pediatrics, Government Medical College, Nagpur, IND

**Keywords:** diagnostic biomarkers, exudative pleural effusion, lactate metabolism, parapneumonic pleural effusion, pleural fluid analysis, pleural fluid lactate, pleural fluid-to-serum lactate ratio, pleural infection, point-of-care testing, tuberculous pleural effusion

## Abstract

Background: Exudative pleural effusion presents a frequent diagnostic dilemma in clinical practice, particularly in regions where tuberculosis is highly prevalent. Although Light’s criteria, adenosine deaminase (ADA), and cytological analysis are routinely used, their diagnostic performance is limited by issues related to specificity, sensitivity, and processing time. Recently, pleural fluid lactate and the pleural fluid-to-serum (P/S) lactate ratio have been investigated as adjunctive biochemical markers for etiological differentiation.

Objectives: The primary objectives of this study were to evaluate pleural fluid lactate levels and to assess the diagnostic performance of the P/S lactate ratio in patients with exudative pleural effusion. Additionally, the study aimed to determine the utility of these biomarkers in differentiating tubercular, malignant, parapneumonic, and other etiologies using receiver operating characteristic (ROC) curve analysis.

Methods: This cross-sectional observational study included 50 adult patients diagnosed with exudative pleural effusion according to Light’s criteria. Based on clinical, radiological, cytological, and microbiological findings, patients were categorized into tubercular, malignant, parapneumonic, and miscellaneous groups. Pleural fluid and serum lactate levels were measured using a point-of-care blood gas analyzer, and the P/S lactate ratio was calculated. Diagnostic performance was analyzed using ROC curves.

Results: Tubercular effusion was identified in 27 patients (54%), malignant effusion in 13 (26%), parapneumonic effusion in seven (14%), and miscellaneous causes in three (6%). Median pleural fluid lactate levels were highest in parapneumonic effusions (7.6 mmol/L; IQR 7.2-8.2), followed by tubercular (4.5 mmol/L; IQR 4.2-5.2) and malignant effusions (1.82 mmol/L; IQR 1.5-7.5) (p<0.01). A pleural lactate cut-off value >7 mmol/L effectively identified parapneumonic/complicated effusions. The mean P/S lactate ratio was significantly higher in tubercular effusions compared to non-tubercular effusions (5.29±2.17 vs. 3.20±2.34; p<0.01).

Conclusion: Pleural fluid lactate and the P/S lactate ratio offer useful adjunctive information in the evaluation of exudative pleural effusions. While absolute lactate values assist in identifying parapneumonic effusions, the P/S ratio provides additional value in differentiating tubercular from non-tubercular causes. These parameters may improve diagnostic efficiency, particularly in resource-constrained settings.

## Introduction

Pleural effusion refers to the unusual accumulation of fluid in the pleural cavity and is a commonly encountered clinical condition worldwide. Pleural effusions are broadly classified into transudative and exudative types based on the underlying pathophysiological mechanism. Transudative effusions are usually associated with overall health issues, like congestive heart failure, liver cirrhosis, or nephrotic syndrome, and usually require only minimal assessment. In contrast, exudative pleural effusions necessitate comprehensive investigation to determine the underlying etiology, including tuberculosis, malignancy, parapneumonic effusion, and less common causes such as connective tissue disorders, pulmonary embolism, complicated pancreatitis, and uremic pleurisy [1].

In regions where tuberculosis is prevalent, such as India, tuberculous pleural effusion is the leading cause of exudative pleural effusion. In contrast, malignant and parapneumonic effusions are more commonly observed in developed countries [2]. Light’s criteria continue to be widely used to differentiate transudative from exudative pleural effusions because of their high sensitivity; however, they lack specificity in identifying the precise etiology of exudative effusions [3].

Routine pleural fluid investigations, including glucose, pH, and lactate dehydrogenase (LDH), along with cytological and microbiological analyses, provide valuable diagnostic information but are associated with notable limitations such as false-negative results, variable specificity, delayed turnaround times, and reliance on specialized laboratory facilities [4,5]. These limitations are especially important in cases of parapneumonic and complicated pleural effusions, where quick diagnosis and timely treatment, such as initiating appropriate antibiotics or draining pleural fluid, are crucial for reducing morbidity and mortality [6].

Adenosine deaminase (ADA) is widely used as a diagnostic biomarker in tuberculous pleural effusion; however, its sensitivity and specificity vary depending on disease prevalence and clinical context [7].

Increased metabolic activity of leukocytes and microorganisms within the pleural space promotes anaerobic glycolysis, resulting in the accumulation of lactic acid. Pleural fluid lactate indicates local hypoxia and inflammatory activity, positioning it as a potential biomarker in pleural effusion analysis. Elevated pleural fluid lactate levels have been reported in infectious pleural conditions, including parapneumonic effusion, complicated pleural effusion, and tuberculous pleuritis [8,9]. Malignant pleural effusions may also demonstrate increased lactate concentrations due to altered tumor metabolism, although their diagnostic utility remains inconsistent [5].

Point-of-care lactate estimation using blood gas analyzers allows rapid and cost-effective testing with minimal sample volume, making it particularly useful in emergency and resource-limited healthcare settings [9]. Furthermore, ratio-based pleural biomarkers have demonstrated improved discriminatory ability in differentiating tubercular from non-tubercular effusions [10].

Consequently, this study was conducted to assess pleural fluid lactate levels and the pleural fluid-to-serum (P/S) lactate ratio in patients with exudative pleural effusions, as well as to evaluate their diagnostic efficacy in distinguishing tubercular, malignant, parapneumonic, and other etiologies.

## Materials and methods

Study design and setting

This study was designed as a cross-sectional observational analysis carried out within the Department of Medicine at a tertiary care hospital in India, spanning a duration of 12 months from 2024 to 2025. The research received approval from the Institutional Ethics Committee, and all participants provided written informed consent prior to enrollment.

Study population

The research included 50 adult patients (≥18 years) who were diagnosed with exudative pleural effusion according to Light’s criteria. Light’s criteria classify pleural effusion as exudative when at least one of the following conditions is met: a pleural fluid protein-to-serum protein ratio exceeding 0.5, a pleural fluid LDH-to-serum LDH ratio greater than 0.6, or a pleural fluid LDH level that is more than two-thirds of the upper limit of normal serum LDH. Patients who were pregnant or those with frank pus on thoracocentesis were excluded. Given that this investigation was exploratory and conducted at a single center, a formal sample size calculation was not performed; instead, the sample size was based on convenience. Therefore, all eligible patients who participated during the study period were included. Demographic details, presenting symptoms, comorbidities, smoking and alcohol history, and clinical examination findings were recorded for analysis.

Pleural fluid collection

Ultrasound-guided thoracocentesis was performed under strict aseptic precautions. Approximately 20-30 mL of pleural fluid was collected before initiation of any antimicrobial therapy. Simultaneously, 5 mL of venous blood was obtained for serum biochemical analysis.

Laboratory analysis

Pleural fluid samples were divided and analyzed as follows: biochemical analysis included LDH, glucose, ADA, and protein; cytological analysis included total leukocyte count, differential leukocyte count, and malignant cytology; microbiological analysis included bacterial culture and cartridge-based nucleic acid amplification test (CBNAAT); and blood gas analysis included pleural fluid pH and lactate. Blood tests were performed as needed and included a complete blood count, liver function tests, kidney function tests, procalcitonin, B-type natriuretic peptide, and tumor markers. Radiological investigations such as chest radiography, computed tomography of the chest, and thoracic ultrasonography were performed on a case-by-case basis.

Objectives

The primary objectives of this study were to evaluate pleural fluid lactate levels in patients with exudative pleural effusion and to assess the diagnostic performance of the P/S lactate ratio in differentiating various etiologies of exudative pleural effusion. The secondary objective was to compare the diagnostic accuracy of these biomarkers in distinguishing tubercular, malignant, parapneumonic, and other causes using receiver operating characteristic (ROC) curve analysis.

Study tools

Pleural fluid and serum lactate levels were measured immediately after sample collection using a point-of-care blood gas analyzer (Radiometer ABL800 FLEX analyzer). The analyzer was calibrated according to manufacturer guidelines, and internal quality control procedures were followed. Lactate estimation (mmol/L) was completed within 10 minutes after collection under standardized conditions to minimize pre-analytical variability. The P/S lactate ratio was calculated for each patient. Pleural fluid ADA levels were measured using a spectrophotometric kit-based method.

Definitions

Etiological diagnosis was established based on a combination of clinical presentation, radiological findings, cytological examination, microbiological testing, and biochemical parameters. a) Tuberculous pleural effusion was diagnosed based on clinical features, radiological findings, and pleural fluid analysis, including ADA >40 U/L. Microbiological confirmation (smear, culture, or CBNAAT) was considered where available. b) Malignant pleural effusion was confirmed by cytological or histopathological evidence. In cases without confirmation, the diagnosis was based on imaging findings suggestive of malignancy and clinical correlation. c) Parapneumonic effusion refers to pleural effusion linked to pneumonia or lung abscess, characterized by fever, leukocytosis, and radiological consolidation. d) Miscellaneous effusions included effusions due to connective tissue disorders, pulmonary embolism, or other less common causes such as uremic pleurisy and complicated pancreatitis.

Statistical analysis

The data were entered into Microsoft Excel and subsequently analyzed using SPSS version 25 (IBM Corp., Armonk, NY, USA). Normality of variables was assessed using the Kolmogorov-Smirnov and Shapiro-Wilk tests. Quantitative variables were expressed as mean±standard deviation or median with IQR, as appropriate. Group comparisons were performed using the Kruskal-Wallis test based on data distribution. ROC curve analysis was performed to assess diagnostic performance. Correlations among quantitative variables were evaluated using Pearson or Spearman correlation coefficients. A p-value <0.05 was considered statistically significant.

Ethical considerations

All participants were provided with a detailed explanation of the study’s objectives and procedures before enrollment. All patients provided written informed consent. Participation in the study was entirely voluntary, allowing participants to withdraw at any point without any negative consequences. The study ensured the protection of patient information at all times.

## Results

The study involved a group of 50 patients diagnosed with exudative pleural effusions, with a mean age of 38.5±14.4 years. The group exhibited a slight male dominance, with males accounting for 56% of the overall population. The main clinical symptoms observed were cough (92%), fever (82%), sputum production (82%), and shortness of breath (76%). Diabetes mellitus was found to be the leading comorbidity, affecting 42% of the population, whereas hypertension was observed in 32% of cases. Radiological assessments indicated that pleural effusions were primarily located on the left side, accounting for 64% of cases, while right-sided effusions represented 22%, and bilateral effusions constituted 14%.

In terms of etiology, tuberculous pleural effusions constituted 54% (27 out of 50) of the cases, whereas malignant effusions made up 26% (13 out of 50). Parapneumonic effusions comprised 14% (7 of 50), while miscellaneous causes represented 6% (3 of 50).

The measured levels of lactate in pleural fluid exhibited substantial variations based on the underlying etiology. The highest median lactate levels were noted in cases of parapneumonic effusions, with a median lactate level of 7.6 mmol/L, falling within a range of 7.2-8.2. The median lactate level in tubercular effusions was found to be 4.5 mmol/L, with values ranging from 4.2 to 5.2. Malignant effusions showed the lowest median pleural lactate concentration, measured at 1.82 mmol/L, with a range from 1.5 to 7.5 mmol/L. The differences observed were statistically significant (p<0.01).

A significant variation was observed in the ratio of P/S lactate among the groups. The mean ratio calculated for tubercular effusions was found to be 5.29±2.17, while non-tubercular effusions showed a mean ratio of 3.20±2.34 (p<0.01). This finding indicates more pronounced metabolic changes linked to tuberculosis.

Compared to other groups, tubercular effusions had considerably higher levels of ADA, with a median value of 64 U/L. This difference was statistically significant (p<0.001). The accompanying tables (Table 1 and Table 2) provide a clear presentation of the demographic and clinical characteristics, along with the biochemical parameters of pleural fluid, including lactate, ADA, protein, and the lactate ratio.

**Table 1:** Baseline demographic and clinical characteristics of patients with exudative pleural effusion

Variable	Category	n (%)
Age group (years)	≤20	4 (8.0)
	21-30	15 (30.0)
	31-40	8 (16.0)
	41-50	14 (28.0)
	51-60	3 (6.0)
	>60	6 (12.0)
Gender	Male	28 (56.0)
	Female	22 (44.0)
Presenting symptoms	Cough	46 (92.0)
	Sputum	41 (82.0)
	Fever	46 (82.0)
	Shortness of breath	38 (76.0)
	Chest pain	21 (42.0)
	Orthopnea	9 (18.0)
	Palpitation	3 (6.0)
Side of pleural effusion	Left	32 (64.0)
	Right	11 (22.0)
	Bilateral	7 (14.0)
Comorbidities	Diabetes mellitus	21 (42.0)
	Hypertension	16 (32.0)
	Smoking	13 (26.0)
	Thyroid disorder	5 (10.0)
	Immunocompromised state	3 (6.0)
	Obesity	3 (6.0)
	Chronic liver disease	3 (6.0)
	Coronary artery disease	2 (4.0)
	Chronic kidney disease	2 (4.0)

**Table 2:** Pleural fluid analysis in various etiologies of exudative pleural effusion in study subjects (n=50) LDH, lactate dehydrogenase; ADA, adenosine deaminase; P/S, pleural fluid-to-serum; ANOVA, analysis of variance

	Tubercular (n=27)	Malignant (n=13)	Parapneumonic (n=7)	Miscellaneous (n=3)	P-value	Test used	One-way ANOVA H-test value/Kruskal-Wallis H-test value
pH	7.33±0.12	7.36±0.13	7.26±0.06	7.16±0.11	0.04	One-way ANOVA	2.96
Pleural lactate (mmol/L)	4.5 (4.2-5.2)	1.82 (1.5-7.5)	7.6 (7.2-8.2)	6	<0.01	Kruskal-Wallis	13.06
Serum lactate (mmol/L)	0.9 (0.8-1.0)	2.2 (0.85-2.60)	3.2 (1.8-4.2)	0.9	0.001	Kruskal-Wallis	15.43
Sugar (mg/dL)	46 (20-98)	84 (50-101)	31 (20-42)	71	0.08	Kruskal-Wallis	6.62
Protein (gm/dL)	4.97±0.70	3.66±0.78	4.24±1.15	4.83±1.22	0.001	One-way ANOVA	7.87
LDH (U/L)	1240 (899-1480)	1180 (499-1400)	1193 (660-1260)	560	0.62	Kruskal-Wallis	1.73
ADA (U/L)	64 (52-68.2)	26 (20.15-33)	38 (22.4-48)	35	<0.001	Kruskal-Wallis	24.39
P/S lactate ratio	5.29±2.17	2.34±1.61	3.17±2.28	7.01±1.61	<0.01	One-way ANOVA	7.22

Analysis of the ROC curve

**Figure 1:**
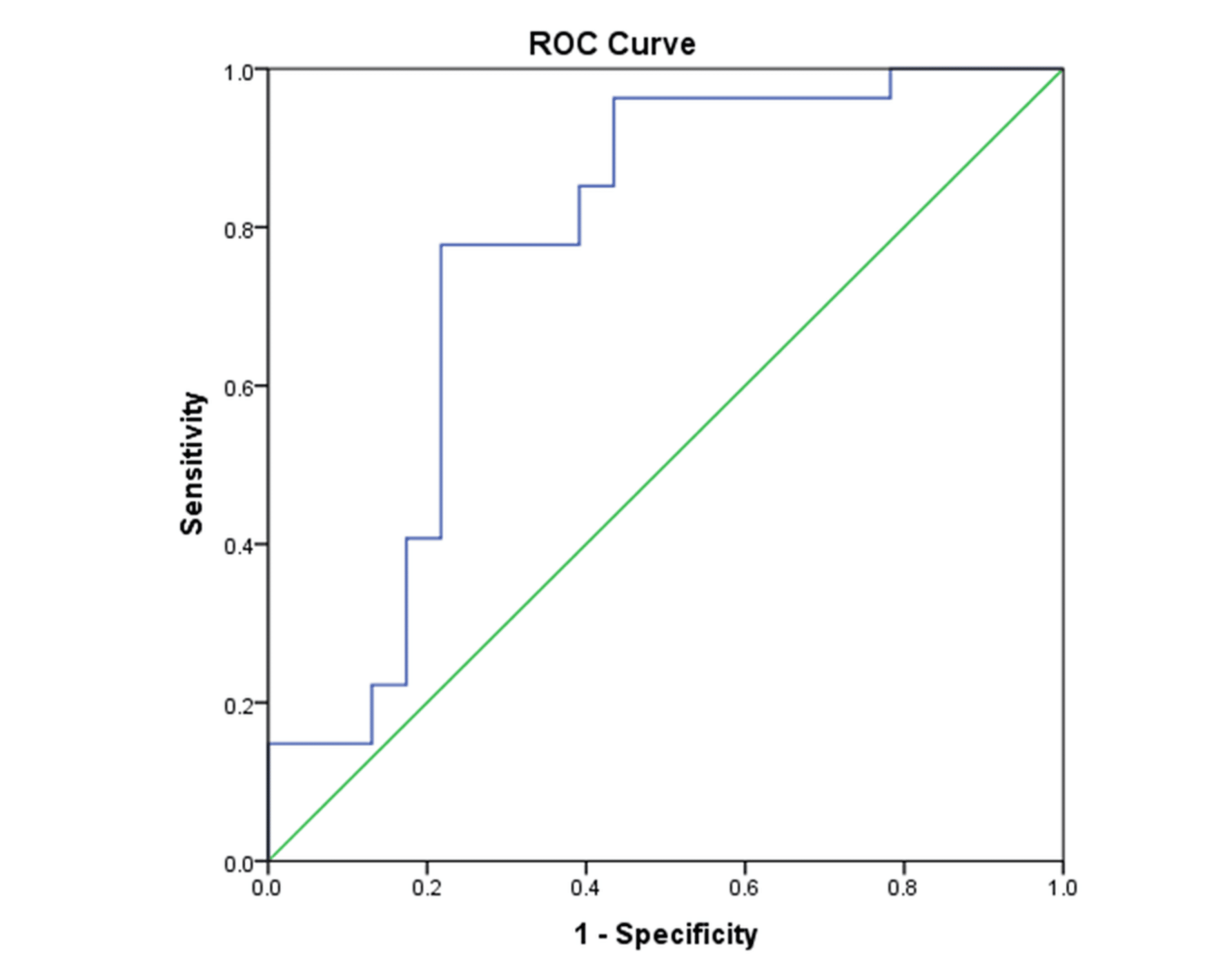
The ROC curve illustrates the diagnostic efficacy of the P/S lactate ratio in distinguishing between tubercular and non-tubercular pleural effusions The AUC value recorded was 0.77, accompanied by a 95% CI ranging from 0.63 to 0.91. P/S, pleural fluid-to-serum; ROC, receiver operating characteristic; AUC, area under the curve

Analysis of the ROC curve (Figure 1) demonstrated that the ratio of P/S lactate exhibited strong diagnostic potential in distinguishing tubercular pleural effusion from non-tubercular pleural effusions, achieving an area under the curve (AUC) of 0.77 (95% CI 0.63-0.91; p=0.001). A cutoff value of 4.19 yielded a sensitivity of 77.78% and a specificity of 78.26%. The positive likelihood ratio was 3.58, while the negative likelihood ratio was 0.28, resulting in an overall diagnostic accuracy of 78%.

**Figure 2:**
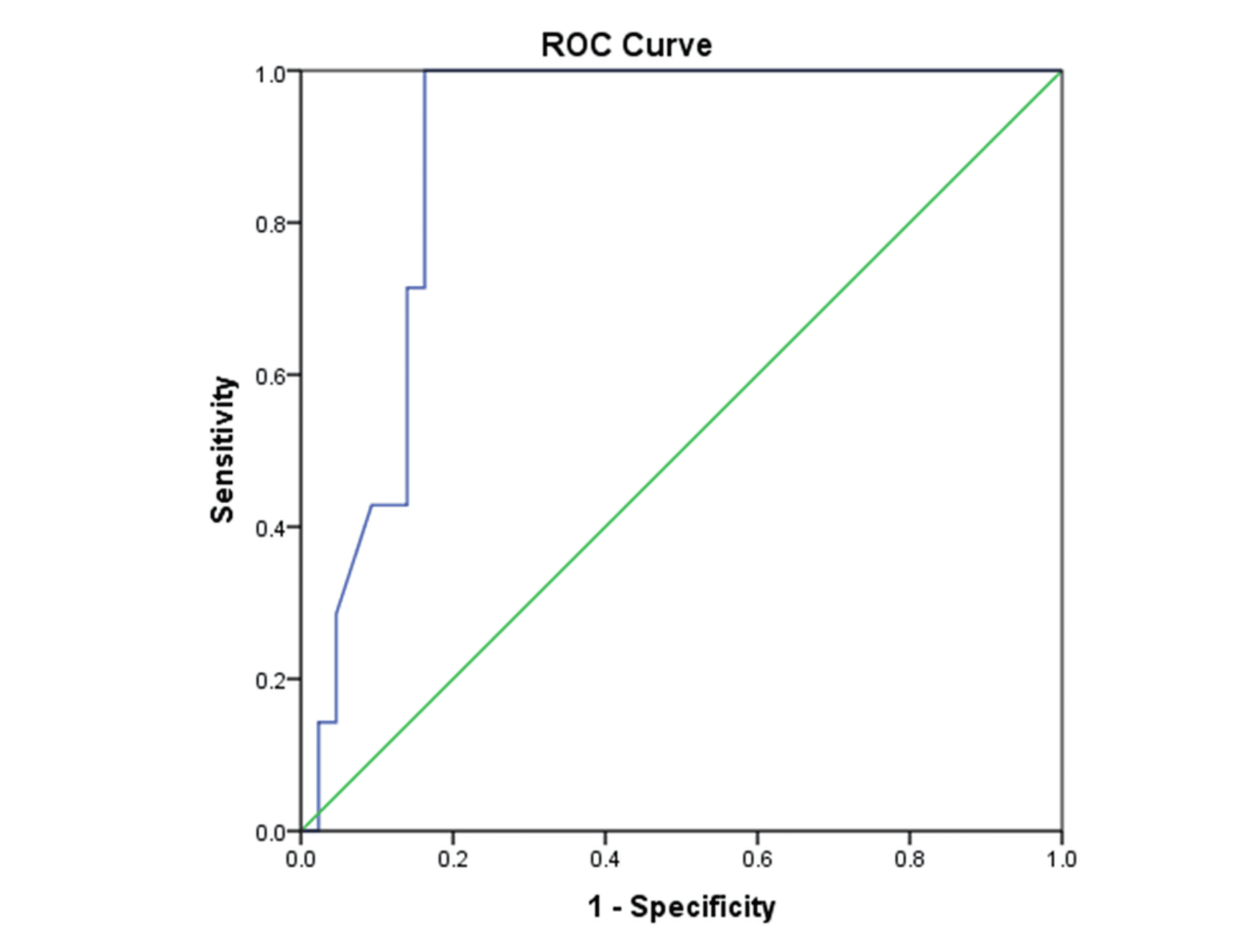
The ROC curve illustrates the diagnostic accuracy of pleural fluid lactate in distinguishing parapneumonic pleural effusion from alternative causes The AUC value recorded was 0.89, accompanied by a 95% CI ranging from 0.80 to 0.98. ROC, receiver operating characteristic; AUC, area under the curve

Analysis of the ROC curve (Figure 2) for pleural fluid lactate demonstrated excellent diagnostic ability in distinguishing parapneumonic pleural effusion from alternative causes, achieving an AUC of 0.89, with a 95% CI ranging from 0.80 to 0.98 and a p-value of 0.001. A cutoff value was established at 7 mmol/L; pleural fluid lactate levels showed a sensitivity of 100% and a specificity of 83.72%. This resulted in a positive likelihood ratio of 6.14 and a negative likelihood ratio of 0.00. The total diagnostic accuracy achieved was 86%.

**Figure 3:**
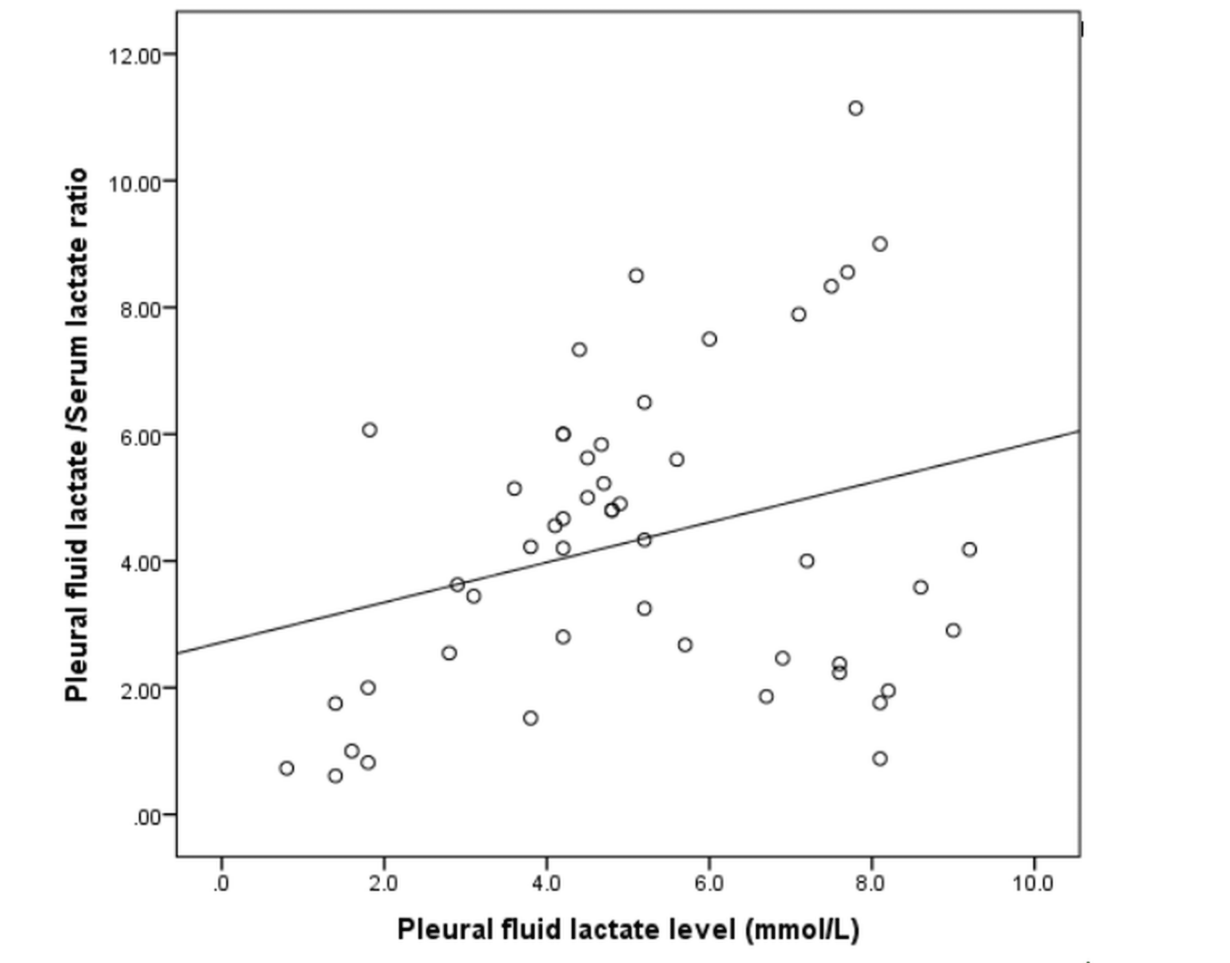
Scatterplot showing correlation of pleural fluid lactate with the P/S lactate ratio in study subjects Correlation analysis was performed using Spearman’s rank correlation coefficient. r-value: 0.28, p-value: 0.04. P/S, pleural fluid-to-serum

Spearman’s rank correlation coefficient was used to evaluate relationships between quantitative variables. A p-value <0.05 was considered statistically significant. A weak positive correlation was observed between pleural fluid lactate levels and the P/S lactate ratio (Table 3 and Figure 3).

**Table 3:** Correlation of pleural fluid lactate with the P/S lactate ratio in study subjects (n=50) P/S, pleural fluid-to-serum

	r-value	P-value
Correlation between pleural fluid lactate and P/S lactate ratio	0.28	0.04

## Discussion

This observational study conducted a cross-sectional analysis involving 50 patients diagnosed with exudative pleural effusion, with a slight male predominance (56%). Tuberculosis was the predominant etiology, identified in 27 patients (54%), followed by malignant pleural effusion in 13 patients (26%), parapneumonic effusion in 7 patients (14%), and miscellaneous causes in 3 patients (6%). These findings reaffirm the persistent burden of tuberculosis as a major cause of exudative pleural effusion in endemic regions such as India.

Pleural fluid lactate levels exhibited considerable variability among various etiologies (p<0.01). Parapneumonic effusions showed the highest lactate levels, with a median value of 7.6 mmol/L (IQR 7.2-8.2). This observation is consistent with earlier studies that reported markedly elevated pleural lactate levels in bacterial pleural infections due to increased anaerobic metabolism, intense inflammatory activity, and impaired pleural perfusion [11-13]. All complicated parapneumonic effusions exceeded the commonly used cut-off values, further supporting the role of pleural lactate as an early indicator of infection severity and complexity.

Tuberculous pleural effusions exhibited moderately elevated pleural lactate levels, with a median of 4.5 mmol/L (IQR 4.2-5.2), which were significantly higher than those observed in malignant effusions. This modest elevation is likely attributable to increased metabolic activity of activated macrophages and lymphocytes within the pleural space in tuberculous pleuritis. However, the observed overlap between tuberculous and malignant effusions limits the utility of pleural lactate as a standalone diagnostic marker for differentiating between these two etiologies.

Malignant pleural effusions demonstrated the lowest median pleural lactate levels at 1.82 mmol/L (IQR 1.5-7.5), although substantial variability was noted. This heterogeneity may reflect differences in tumor burden, metabolic activity, and underlying tumor biology, indicating that pleural lactate alone is not a reliable marker for malignancy, as also reported in prior studies [14].

The P/S lactate ratio showed greater discriminatory capacity in differentiating tubercular from non-tubercular effusions compared with absolute pleural lactate levels. Tubercular effusions had a significantly higher mean ratio (5.29±2.17) than non-tubercular effusions (3.20±2.34; p<0.01), suggesting pronounced compartmentalized metabolic activity in tuberculosis. Similar ratio-based approaches using pleural biomarkers have previously been shown to enhance etiological differentiation, particularly in tuberculosis-endemic settings [14,15]. In this context, pleural lactate parameters may serve as a useful adjunct to ADA, especially in diagnostically challenging cases where conventional markers yield equivocal results.

ROC curve analysis further supported these findings. The P/S lactate ratio demonstrated good diagnostic performance in distinguishing tubercular from non-tubercular pleural effusions (AUC=0.77), with balanced sensitivity and specificity at the selected cut-off. In contrast, pleural fluid lactate showed excellent diagnostic accuracy for parapneumonic effusions (AUC=0.89), achieving 100% sensitivity at the defined threshold, thereby providing a strong rule-out value for non-infectious causes [11-13].

The clinical utility of pleural lactate measurement lies in its rapid availability at the bedside using blood gas analyzers, minimal sample volume requirement, and short turnaround time, making it particularly advantageous in emergency and resource-limited settings [12,15]. Nevertheless, pleural lactate should be interpreted in conjunction with established diagnostic markers such as ADA, cytology, and microbiological analysis, rather than as an independent diagnostic test [14,16].

Despite certain limitations, such as a comparatively small sample size, single-center methodology, and reliance on radiological criteria in selected cases, the consistent and statistically significant differences observed across etiologies highlight the potential role of pleural fluid lactate and its serum ratio as valuable adjunctive diagnostic biomarkers in the evaluation of exudative pleural effusions. The observed differences in pleural fluid lactate levels among various etiologies should be interpreted with caution due to the relatively small sample size and overlap between groups. Larger, multicenter studies are needed to validate these findings.

## Conclusions

Pleural fluid lactate levels were highest in parapneumonic pleural effusions, while tuberculous effusions demonstrated moderately elevated levels, and malignant effusions showed comparatively lower concentrations. A pleural fluid lactate cutoff value >7 mmol/L effectively identified parapneumonic pleural effusions, whereas an elevated P/S lactate ratio showed a strong association with tuberculous pleural effusions.

Incorporating bedside pleural lactate measurement and the P/S lactate ratio into standard pleural fluid analysis could enhance diagnostic precision and expedite clinical decision-making, especially in resource-constrained and tuberculosis-prevalent environments. Although these findings are encouraging, validation through larger, multicenter studies is required to establish standardized cutoff values and strengthen the evidence for routine clinical adoption. Incorporation of pleural lactate testing into diagnostic protocols has the potential to enhance patient management and outcomes.
